# Hypoxia Improves Endurance Performance by Enhancing Short Chain Fatty Acids Production *via* Gut Microbiota Remodeling

**DOI:** 10.3389/fmicb.2021.820691

**Published:** 2022-02-07

**Authors:** Li Huang, Tianyou Li, Min Zhou, Mengyan Deng, Lidong Zhang, Long Yi, Jundong Zhu, Xiaohui Zhu, Mantian Mi

**Affiliations:** Chongqing Key Laboratory of Nutrition and Food Safety, Research Center for Nutrition and Food Safety, Chongqing Medical Nutrition Research Center, Institute of Military Preventive Medicine, Third Military Medical University, Chongqing, China

**Keywords:** endurance performance, gut microbiota, hypoxia, short chain fatty acids, mitochondrial biogenesis

## Abstract

Hypoxia environment has been widely used to promote exercise capacity. However, the underlying mechanisms still need to be further elucidated. In this study, mice were exposed to the normoxia environment (21% O_2_) or hypoxia environment (16.4% O_2_) for 4 weeks. Hypoxia-induced gut microbiota remodeling characterized by the increased abundance of *Akkermansia* and *Bacteroidetes* genera, and their related short-chain fatty acids (SCFAs) production. It was observed that hypoxia markedly improved endurance by significantly prolonging the exhaustive running time, promoting mitochondrial biogenesis, and ameliorating exercise fatigue biochemical parameters, including urea nitrogen, creatine kinase, and lactic acid, which were correlated with the concentrations of SCFAs. Additionally, the antibiotics experiment partially inhibited hypoxia-induced mitochondrial synthesis. The microbiota transplantation experiment demonstrated that the enhancement of endurance capacity induced by hypoxia was transferable, indicating that the beneficial effects of hypoxia on exercise performance were partly dependent on the gut microbiota. We further identified that acetate and butyrate, but not propionate, stimulated mitochondrial biogenesis and promoted endurance performance. Our results suggested that hypoxia exposure promoted endurance capacity partially by the increased production of SCFAs derived from gut microbiota remodeling.

## Introduction

There are many benefits of hypoxia, such as helping weight loss, maintaining cardio-metabolic health (Kayser and Verges, 2013; Woolcott et al., 2015; Hobbins et al., 2017), as well as improving exercise performance (Girard et al., 2013a,b; Wang et al., 2019). Exercise training under hypoxic conditions has been widely accepted as a mean to enhance athletic endurance ability in the past few decades (Girard et al., 2013a; Millet and Brocherie, 2020). The predominant mechanisms of hypoxic training on improving sports performance focused on hematological factors such as increased red blood cell (RBC) counts and high hemoglobin (HGB) concentrations, as well as non-hematological factors including running economy, lactate threshold, mitochondrial gene expression, and enhanced muscle buffering capacity (Saunders et al., 2013; Mujika et al., 2019). However, further researches are needed to clarify the other mechanisms underlying hypoxia increasing exercise performance.

The gut microbiota and its hosts maintain a mutually beneficial relationship and dynamic coexistence (Fan and Pedersen, 2021). Recent evidence indicated that the gut microbiota plays a significant role in exercise performance (Lahiri et al., 2019; Mailing et al., 2019; Nay et al., 2019; Scheiman et al., 2019). Compared with specific pathogen-free mice, germ-free mice showed skeletal muscle atrophy and lower endurance capacity, while recovering the gut microbiota of germ-free mice resulted in improved endurance performance and skeletal muscle mass increasing (Hsu et al., 2015; Huang et al., 2019; Lahiri et al., 2019). *Veillonella*, enriched in marathon runners, could metabolize lactate into acetate and propionate and converse inorganic nitrate to nitric oxide and related nitrogen oxides, thus improving exercise performance (Scheiman et al., 2019; Lundberg et al., 2021). Moreover, short-chain fatty acids (SCFAs) produced by the gut microbiota are now recognized as the potential regulators of skeletal muscle metabolism and function to enhance exercise performance (Okamoto et al., 2019; Frampton et al., 2020; Hu et al., 2020; Bongiovanni et al., 2021). These findings indicated that the gut microbiota might improve exercise performance through its derived metabolites participating in host metabolism.

In recent years, the effects of hypoxia on the gut microbiota attracted much attention (Mazel, 2019; Han et al., 2021). Short-term or chronic exposure to hypoxia can influence the composition and diversity of the gut microbiota (Li and Zhao, 2015; Karl et al., 2018; Suzuki et al., 2019; Jia et al., 2020), thus helping the hosts to adapt to the environment (Li et al., 2016, 2018, 2019; Sun et al., 2019; Ma et al., 2021). Current findings indicated that hypoxia exposure leads to intestinal hypoxia, thus promoting the growth of anaerobic bacteria (Suzuki et al., 2019; Jia et al., 2020). However, the interventions considerably varied with regards to the hypoxia model such as exposure time and oxygen concentration, and to the confounders such as exercise or diet. Thus, the influences of hypoxia exposure on the gut microbiota are still controversial (Li and Zhao, 2015; Karl et al., 2018; Suzuki et al., 2019; Han et al., 2020, 2021; Jia et al., 2020).

Hence, we aimed to investigate the mechanisms underlying hypoxia exposure enhancing endurance performance. Interestingly, we found that hypoxia significantly improved endurance performance by increasing the abundance of the *Akkermansia* and *Bacteroides* genus. Further results revealed that the SCFAs produced by the gut microbiota enhanced mitochondrial biogenesis, thus improving endurance capacity. Our results suggested that hypoxia promotes exercise performance through the regulation of the gut microbiota-SCFAs axis.

## Materials and Methods

### Animals

Healthy male C57BL/6J mice (6–8 weeks old) were acquired from the Animal Experimental Center of Third Military Medical University (Chongqing, China). Animals were housed under a 12-h light/dark cycle with *ad libitum* access to food and water. During the experiments, body weight was recorded weekly. Before the experiments, mice were placed in a motorized treadmill (SANS Biological Technology, China) and ran at 15 m/min speed for 10 min once a day for 5 days as acclimatized training. At the end of the experiments, mice ran at the speed of 25 m/min at normobaric normoxia until exhaustion as endurance test. In the acclimatized training and the endurance test, the acceleration was 5 m/min^2^, and timing started when the speed reached 15 and 25 m/min, respectively. The current of electrode slice was set to 2 mA to prod mice to keep running. In the endurance test, exhaustion was defined as the inability to run for 20 s under the prodded electrode slice, as we described before (Hou et al., 2020). After mice exhaustion, blood samples were taken from the tail vein to measure blood glucose and lactic acid with the OneTouch blood glucose meter (LifeScan, United States) and Lactate Scout (EKF, Germany), respectively. Then mice were sacrificed immediately. Soleus and gastrocnemius muscle mass were examined by an electronic scale and stored at −80°C. The Animal Care and Use Committee of the Third Military Medical University approved all the animal experiments.

### Experimental Design

The experimental design shown in Supplementary Figure 1.

#### Animal Experiment 1

To determine the effects of hypoxia on exercise performance and gut microbiota, mice were randomly divided into two groups: normobaric normoxia (Normoxia; 21.0% O_2_) and normobaric hypoxia (Hypoxia; 16.4% O_2_) (*n* = 8/group). Grip strength and hematological parameters were measured at the different time points of 0, 2, and 4w. Stool samples from both groups were collected at the end of treatment (Supplementary Figure 1A).

#### Animal Experiment 2

To identify the role of gut microbiota in hypoxia improving exercise intolerance, we performed antibiotic treatment and fecal microbiota transplantation (FMT) experiments.

a. For the antibiotic treatment experiment, mice were randomly divided into two groups (*n* = 9/group). One group (Hypoxia) was exposed to a hypoxia environment for 4 weeks. The other group (Hypoxia + Abx) was exposed to a hypoxia environment accompanied by 200 μL antibiotic cocktail (Abx) (1 g/L ampicillin, 1 g/L metronidazole, 1 g/L neomycin, 0.5 g/L vancomycin) by gavage for 7 days to clear gut microbiota. During the remaining 3 weeks, mice were treated with 200 μL Abx every other day to keep the flora eliminated (Supplementary Figure 1B; Hui et al., 2020). Mice treated with Abx did not show any illness or distress.

b. Stool samples collected from animal experiment 1 were dissolved in PBS (20 mg/mL), shaken for 3 min, and centrifuged for 3 min. The supernatant was collected and stored at −80°C. The receipt mice were treated with 200 μL Abx by gavage for 1 week. Then, mice were treated with 100 μL of the supernatant once daily for seven consecutive days in the first week of colonization. During the remaining 3 weeks, fecal slurries were introduced every other day to reinforce colonization (*n* = 9/group) (Supplementary Figure 1C).

#### Animal Experiment 3

To identify the important role of SCFAs in hypoxia improving exercise endurance, mice received 150 mM acetate (Sigma, United States), propionate (Sigma, United States), butyrate (Sigma, United States) or NaCl (vehicle) in the drinking water for 4 weeks as reported before (*n* = 9/group) (Supplementary Figure 1D; Smith et al., 2013; Fachi et al., 2019).

### Hypoxia Treatment

An 8 mm thick acrylic cabin (180 × 50 × 50 cm) was used to create a normobaric hypoxia environment. The normobaric hypoxia cabin was designed by Professor Xiaohui Zhu, and produced by TOW-INT Tech (China). Environmental hypoxia was generated by adjusting the relative concentrations of oxygen and nitrogen in the input gas mixture. With constant monitoring, continuous gas flow and CO_2_ absorption by Ca(OH)_2_ within the hypoxic cabin maintained CO_2_ levels below 0.5%. Adjust the O_2_ concentration in the cabin to 16.4% within 30 min, equivalent to an altitude of 2,000 m. A controlled environment (21% O_2_) was created with an identical cabin setup. During the experiment, the temperature was maintained at 23 ± 2°C, and the humidity was 65 ± 5%. Changing food, sawdust, and Ca(OH)_2_ every day from 9:00 to 10:00 at normoxia environment.

### Grip Strength Test

Grip strength was measured by a grid-connected to the grip strength meter (Ugo Basile, Germany). The mice underwent three tests, with at least 10 min recovery time between tests. The mean measure of maximal force (in grams) recorded was used as the grip strength.

### Hematological Parameters Measurement

Blood was collected from the tail veins of mice and diluted in 1% EDTA solution. Red blood cell (RBC) counts and hemoglobin (HGB) concentration were measured automatically by a hematology analyzer (XT-1800i/2000IV; Sysmex, Kobe, Japan).

### Biochemical Parameters Measurement

Serum was obtained from blood samples in mice, and biochemical profiles including creatine kinase (CK) and urea nitrogen (BUN) were measured with commercial kits (Solarbio, China). Hepatic glycogen was determined using Glycogen Detection Kit (Solarbio, China) according to the manufacturer’s instruction. All the experiments were tested at least three times.

### Transmission Electron Microscopy Observation

Mitochondrial morphology and number in the gastrocnemius muscle were analyzed by TEM images. Briefly, gastrocnemius was treated and visualized on JEM-1400 microscope (Jeol, Japan) as we described before (Liu et al., 2020). Three animals per group were and ten images per mouse were used for the quantitative analysis of mitochondria. The number of mitochondria per section of intermyofibrillar mitochondria was quantified. The number of mitochondria was counted on micrographs as previously described (Liu et al., 2020).

### Sequencing of the Gut Microbiota

The bacterial genomic DNA from stool samples was extracted using a DNA Stool kit (TianGen, China). The DNA sequence was amplified using barcoded V3-V4 region primers targeting the bacterial 16S rRNA gene, sequenced on the Illumina MiSeq platform (Illumina, San Diego, CA, United States). Analyses of generated and demultiplexed sequences were performed using the QIIME software (v.1.8.0) (Caporaso et al., 2010). Operational taxonomic units (OTUs) with 97% similarity cutoff were clustered using UPARSE (v.7.1) (Edgar, 2013), and chimeric sequences were identified and removed. The α-diversity indexes were calculated by Mothur (v.1.30.1) (Schloss et al., 2009). Principal coordinate analysis (PCoA) based on the unweighted UniFrac distance was performed using the QIIME software. The discriminated taxa of the gut microbiota profiles in different groups were identified using the R and STAMP (v.2.0.0) (Segata et al., 2011). To determine the microbiota-based biomarkers, the Linear Discriminant Analysis Effect Size (LEfSe) method was used, which indicates the taxa that contribute to the uniqueness of the corresponding groups at a linear discriminant analysis (LDA) score of > 2.0 (false discover rate (FDR) < 0.5).

### Measurement of Cecal Contents Short-Chain Fatty Acids

Fatty acid analysis was conducted using an Agilent 6890N GC system (Agilent Technologies, PA, United States) and performed as previously described (Zhao et al., 2006; Kang et al., 2017). Briefly, cecal contents from each mouse were weighed and homogenized in 1 mL deionized water for 3 min. The pH value of the suspension was adjusted to 2–3, and the suspension was subsequently transferred into a polypropylene tube and centrifuged for 20 min at 3,000 g, yielding a clear supernatant. 2-ethylbutyric acid (TEBA) was used as the internal standard and added into the supernatant at a final concentration of 1 mM.

### Quantitative RT-PCR

Total RNA was extracted from gastrocnemius muscle using Trizol reagent (Invitrogen, United States). The NanoDrop spectrophotometer and agarose gel electrophoresis were used to detect RNA concentration and integrity, respectively. Reverse transcription of mRNA into cDNA was carried out with PrimeScript RT master mix (Takara, Japan). And qPCR was performed using SYBR premix Ex Taq (Takara, Japan) with qTOWER 2.2 (Analytik Jena, Germany). The expression of target genes was normalized to β-actin. The primers sequences are listed in Supplementary Table 1.

### Determination of Mitochondrial DNA Copy Number

mtDNA was extracted from gastrocnemius muscle using Mito DNA Extraction Kit (Genmed Scientifics). Cytochrome c oxidase II (COX2) was used for the quantification of the mtDNA copy number, whereas 18S nuclear gene were used for standardization. The primers sequences are listed in Supplementary Table 1.

### Statistical Analysis

Values are expressed as the mead ± SD and statistical analyses were performed using SPSS statistics 19.0 unless otherwise specified. Statistical analyses were applied using the unpaired 2-tailed Student’s *t*-test (for two groups) and one-way analysis of variance followed by LSD *post hoc* tests (for multiple groups comparisons). Correlation analysis was carried out using the Pearson with R software. In all cases, *p* < 0.05 was considered statistically significant. And asterisks denote statistical significance (ns, no significance; *p < 0.05; ^**^p < 0.01; ^***^p < 0.001).

## Results

### Hypoxia Exposure Improved Endurance Performance of Mice

**FIGURE 1:**
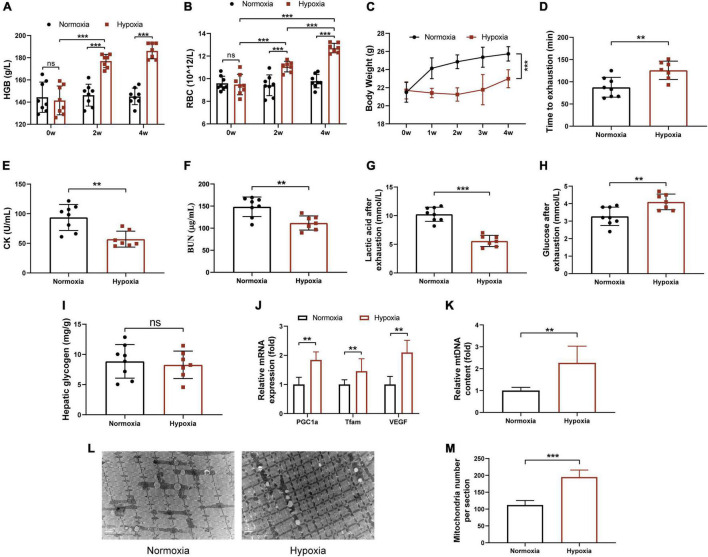
Hypoxia exposure improved endurance performance of mice. HGB **(A)** and RBC **(B)** in the tail blood were detected. **(C)** Body weight was measured once a week. **(D)** Time to exhaustion was recorded. After exhaustion, CK **(E)**, BUN **(F)**, lactic acid **(G)**, and glucose **(H)** were measured immediately. **(I)** The hepatic glycogen was measured. The expression of PGC-1α, Tfam, VEGF **(J)**, and the mtDNA **(K)** content were measured by RT-PCR. **(L)** Representative TEM images of gastrocnemius at a magnification of 12,000×. **(M)** Quantification of the number of mitochondria per section. Data are presented as means ± SD. *p < 0.05, **p < 0.01, ***p < 0.001 (Student’s *t*-test).

To assess the effects of hypoxia on physical performance, mice were exposed to the normoxia environment or the hypoxic environment with 16.4% oxygen content for 4 weeks. As expected, the levels of HGB and RBC of hypoxia group were significantly increased in mice (Figures 1A,B). As shown in Figure 1C, hypoxia exposure significantly reduced body weight of mice, although food intake has no significant difference (Supplementary Figure 2A). Next, we investigated the effects of hypoxia on endurance performance. As shown in Figure 1D, the average time to exhaustion in hypoxia group increased compared with that in normoxia mice. Moreover, hypoxia treatment significantly decreased serum CK, BUN, and blood lactic acid levels, which were used to evaluate the degree of muscle fatigue and muscle damage (Figures 1E–G). In addition, blood glucose of hypoxia group was significantly increased (*p* = 0.0064) compared with normoxia group, and there was no significant difference in hepatic glycogen between the two groups (Figures 1H,I). The RT-PCR analysis indicated that the mRNA expressions of PGC-1α, Tfam, and VEGF of gastrocnemius in hypoxia-exposed mice were significantly increased (Figure 1J). This finding was in line with the quantitative analysis of mtDNA by RT-PCR (Figure 1K) and TEM images (Figures 1L,M). However, we observed that hypoxia exposure did not change grip strength but decreased soleus and gastrocnemius muscles mass (Supplementary Figures 2B–D). Taken together, these results suggested that hypoxia exposure remarkably improved endurance performance but not muscle strength, and promoted mitochondrial biogenesis in skeletal muscle of mice.

### Hypoxia Treatment Modified the Gut Microbiota

**FIGURE 2:**
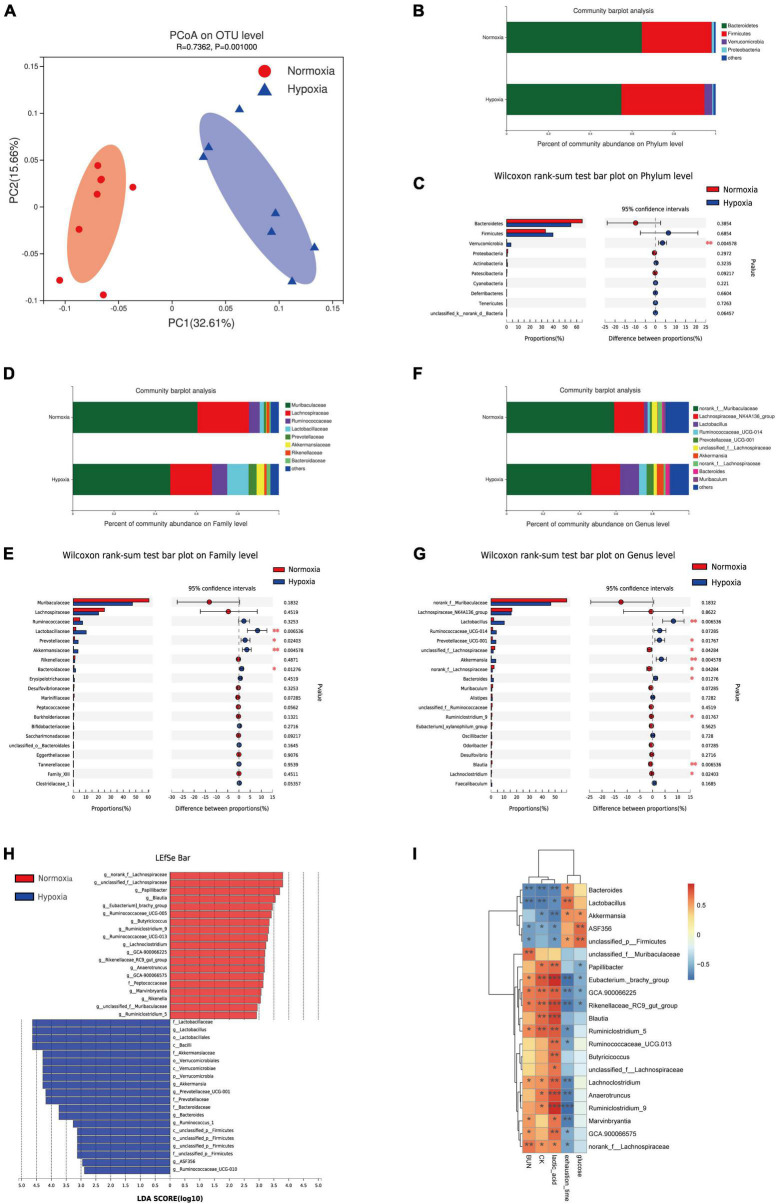
Hypoxia treatment modified the gut microbiota. **(A)** Principal coordinate analysis (PCoA) of the β-diversity based on the unweighted UniFrac distance matrix (at the OTU level). Taxonomic distributions at the phylum level **(B)**, and the Kruskal-Wallis *H* test bar plot at the phylum level **(C)**. Taxonomic distributions at the family level **(D)**, and the Kruskal-Wallis *H* test bar plot at the family level **(E)**. Taxonomic distributions at the genus level **(F)**, and the Kruskal-Wallis *H* test bar plot at the genus level **(G)**. **(H)** Bar graph of linear discriminant analysis (LDA) scores, showing the biomarker taxa (LDA score of > 2 and a significance of *p* < 0.05 determined by the Wilcoxon signed-rank test). **(I)** Heatmap of Pearson’s correlation analysis between the key gut microbial taxa at the genus level and fatigue related factors. *p < 0.05, **p < 0.01, ***p < 0.001.

To investigate the effect of hypoxia exposure on the gut microbiota, fecal samples were harvested at the end of experiment, and the V3 and V4 16S rRNA variable gene regions were amplified and sequenced using the Illumina MiSeq platform. Our result showed no significant effects on α-diversity between the two groups (Supplementary Figures 3A–D). Moreover, a clear separation based on the unweighted UniFrac distances was observed, suggesting that the gut microbiota was substantially remodeled by hypoxia exposure (Figure 2A). Furthermore, at the phylum level, hypoxia treatment markedly increased the abundance of *Verrucomicrobia* (Figures 2B,C), which was related to exercise-driven changes (Kim and Kang, 2019; Zhong et al., 2021). On the family level, we found that hypoxia increased the abundance of the *Lactobacillaceae*, *Prevotellaceae*, *Akkermansiaceae*, and *Bacteroidaceae* (Figures 2D,E). Further analysis at the genus level indicated an increase in the abundance of *Lactobacillus*, *Prevotellaceae_UCG-001*, *Akkermansia*, and *Bacteroides*, whereas reduced *nclassified_f__Lachnospiraceae* and *norank_f__Lachnospiraceae* (Figures 2F,G). The results of the LEfSe indicated that hypoxia caused an increase in the relative abundance of members from the phylum *Verrucomicrobia*, class *Verrucomicrobiae*, family *Prevotellaceae*, and genus *Akkermansia* relative to their levels in the normoxia mice (Figure 2H). Furthermore, the relative abundance of the family *Peptococcaceae* and genus *Blautia* were lower in the hypoxia group (Figure 2H). Further correlation analysis showed that the fecal *Lactobacillus*, *Akkermansia*, and *Bacteroides* abundance were significantly negatively correlated with CK, BUN, and lactic acid levels (Figure 2I and Supplementary Figures 3E–G). The result was in line with some previous studies, which indicated that athletes had a higher abundance of indicated microbiota (Petersen et al., 2017; Huang et al., 2020). Taken together, these results indicated that hypoxia exposure modified gut microbiota composition.

### Microbial Changes Induced by Hypoxia Exposure Regulated Short-Chain Fatty Acids Production

**FIGURE 3:**
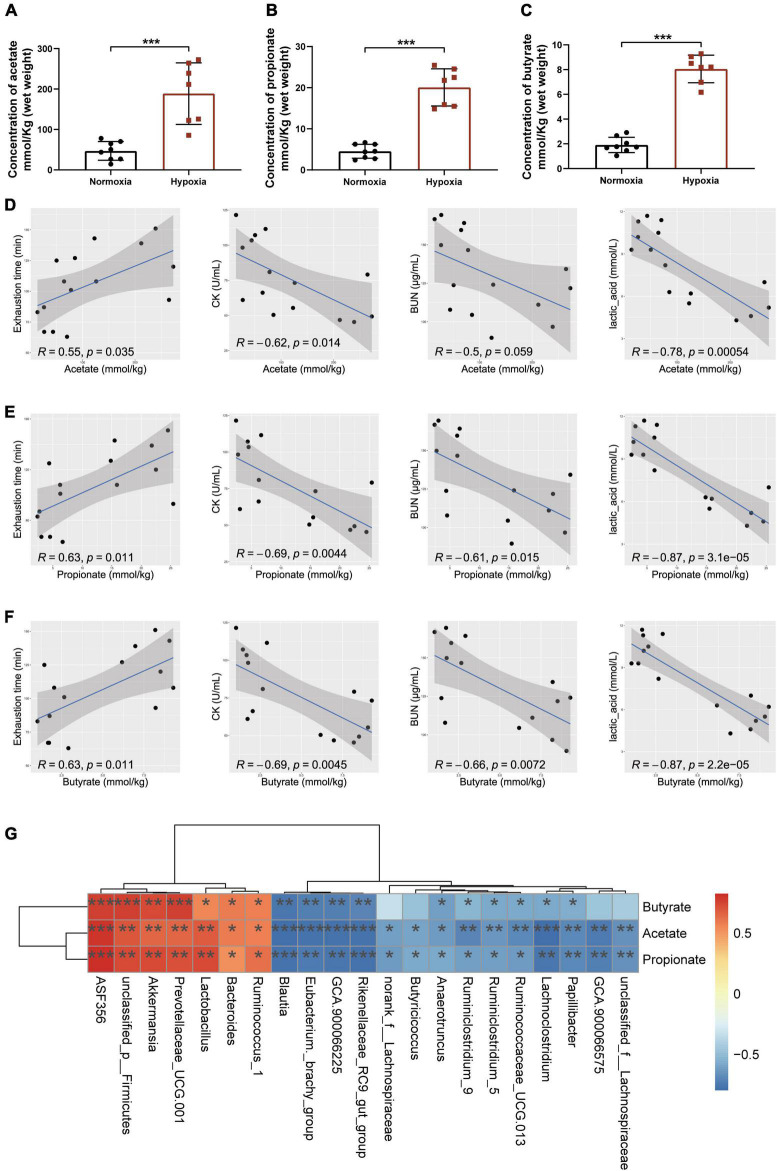
Microbial changes induced by hypoxia exposure regulated SCFAs production. The contents of acetate **(A)**, propionate **(B)**, and butyrate **(C)** were measured by GC–MS in cecal contents. Data are presented as means ± SD. **(D)** Plots show the relationship between the content of acetate and exhaustion time, CK, BUN, and lactic acid, represented as a smoothing spline with a 95% confidence interval (shaded region). **(E)** Plots show the relationship between the content of propionate and exhaustion time, CK, BUN, and lactic acid, represented as a smoothing spline with a 95% confidence interval (shaded region). **(F)** Plots show the relationship between the content of butyrate and exhaustion time, CK, BUN, and lactic acid, represented as a smoothing spline with a 95% confidence interval (shaded region). **(G)** Heatmap of Pearson’s correlation analysis between the key gut microbial taxa at the genus level and the contents of SCFAs in cecal contents. *p < 0.05, **p < 0.01, ***p < 0.001. (**A–C**, Student’s *t*-test; **D–G**, Pearson’s correlation).

As accumulating evidence indicated that SCFAs contributed to exercise endurance (Okamoto et al., 2019; Scheiman et al., 2019; Bongiovanni et al., 2021), we measured the content of SCFAs in cecal contents to determine whether the hypoxia-induced enhancement of endurance capacity could be linked to the changes in SCFAs production. The results showed that hypoxia exposure significantly increased the contents of acetate, propionate, and butyrate (Figures 3A–C). Further correlation analysis showed that the contents of SCFAs were significantly positively correlated with exhaustion time, and negatively correlated with CK, BUN, and lactic acid levels (Figures 3D–F). Interestingly, we found *Akkermansia* and *Bacteroides* were the key microbiota to produce SCFAs (Figure 3G) as reported (Koh et al., 2016). These results indicated that microbial changes induced by hypoxia exposure significantly increased the contents of SCFAs.

### Gut Microbiota Contributed to the Beneficial Effects of Hypoxia on Endurance Performance

**FIGURE 4:**
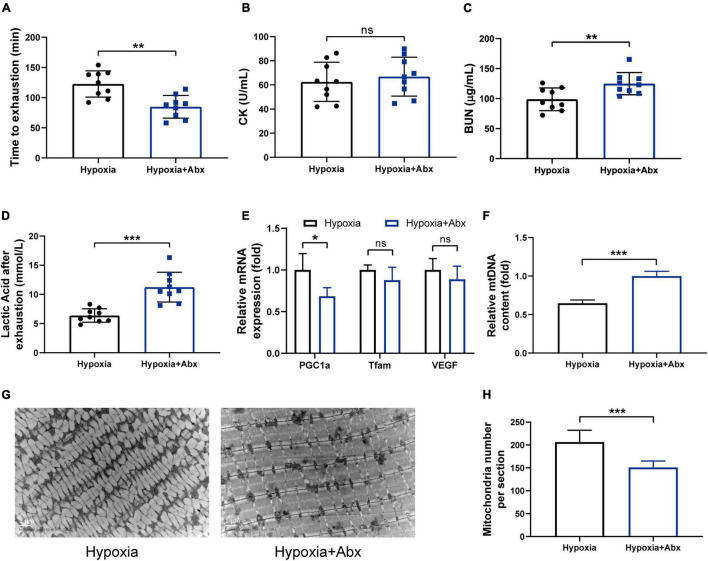
Gut microbiota contributed to the beneficial effects of hypoxia on endurance performance. **(A)** Time until exhaustion was recorded. After exhaustion, CK **(B)**, BUN **(C)**, and lactic acid **(D)** were measured immediately. The expression of mitochondrial biogenesis **(E)** and the mtDNA content **(F)** were measured by RT-PCR. Representative TEM images **(G)** and quantification **(H)** of mitochondria in gastrocnemius muscle at original magnification of ×12,000. Data are presented as means ± SD. *p < 0.05, **p < 0.01, ***p < 0.001 (Student’s *t*-test, ns, no significance).

To determine whether the gut microbiota contributes to the beneficial effects of hypoxia on endurance performance, the experiment including antibiotic treatment and FMT was performed. The results showed that the beneficial effects of hypoxia on endurance performance were inhibited by Abx treatment (Figures 4A–D). Furthermore, we observed that Abx treatment decreased mitochondrial biogenesis (Figures 4E–H). However, Abx treatment had no influence on hematological parameters (Supplementary Figures 4A–C). In addition, Abx treatment reduced the gastrocnemius muscle weight and grip strength (Supplementary Figures 4D–F), which was in line with reported before (Lahiri et al., 2019).

**FIGURE 5:**
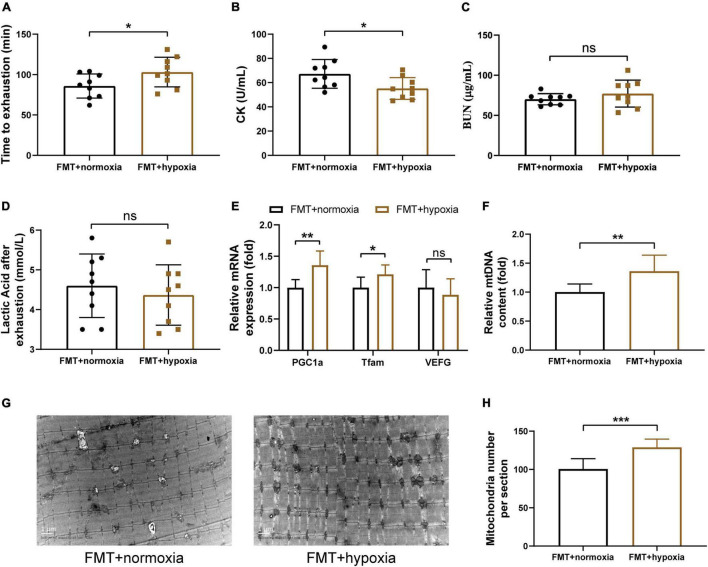
SCFAs supplementation enhanced endurance capacity and promoted mitochondrial biogenesis. **(A)** Running time until exhaustion was recorded. CK **(B)**, BUN **(C)**, and lactic acid **(D)** were measured immediately after exhaustion. The expression of mitochondrial biogenesis **(E)** and the mtDNA content **(F)** were measured by RT-PCR. **(G)** Representative TEM images of gastrocnemius at a magnification of 12,000×. **(H)** Quantification of the number of mitochondria per section. Data are presented as means ± SD. *p < 0.05, **p < 0.01, ***p < 0.001 (Student’s *t*-test, ns, no significance).

To investigate whether the hypoxia-induced gut microbiota shift contributes to the increased endurance capacity and SCFAs concentration, the fecal suspensions derived from the normoxia and hypoxia-treated mice were transplanted into microbiota-depleted mice. We found that the effects of hypoxia on SCFAs concentration were transferable by the gut microbiota (Supplementary Figures 5A–C). Furthermore, transplantation of gut microbiota from the hypoxia-treated mice increased the endurance performance of receipt mice (Figures 5A–D). Moreover, this finding was in line with the increased skeletal muscle mitochondrial biogenesis observed in the receipt mice (Figures 5E–H). However, gut microbiota transplantation had no influence on hematological parameters, muscle weights, and grip strength (Supplementary Figures 5D–I). These results collectively suggested that the beneficial effects of hypoxia on endurance performance and mitochondrial biogenesis were partly dependent on the gut microbiota.

### Short-Chain Fatty Acids Supplementation Enhanced Endurance Capacity and Promoted Mitochondrial Biogenesis

**FIGURE 6:**
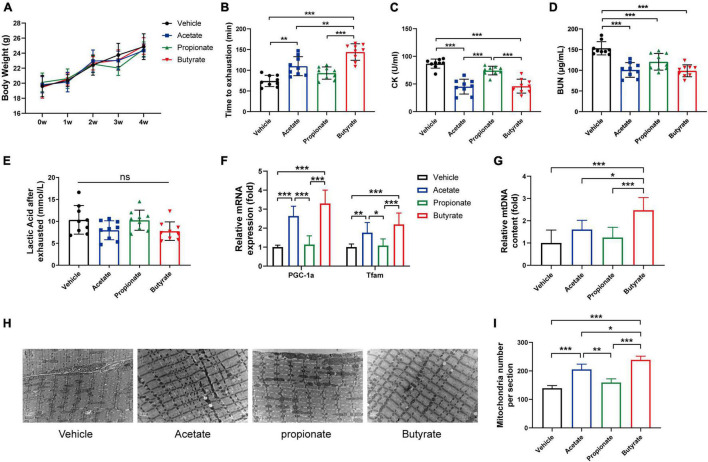
SCFAs supplement enhanced endurance capacity by promoting mitochondrial synthesis. **(A)** Body weight was measured once a week. **(B)** Time to exhaustion was recorded. After exhaustion, CK **(C)**, BUN **(D)**, and lactic acid **(E)** were measured immediately. RT-PCR analysis of mitochondrial biogenesis gene expression **(F)** and mtDNA content **(G)** in gastrocnemius. Representative TEM images **(H)** and quantification **(I)** of mitochondria in gastrocnemius muscle at original magnification of ×12,000. Data are presented as means ± SD. *p < 0.05, **p < 0.01, ***p < 0.001 (One way ANOVA).

Previous studies reported that SCFAs mediate metabolic cross-talk between the gut microbiota and skeletal muscle (Okamoto et al., 2019; Frampton et al., 2020). SCFAs were also found to enhance mitochondrial biogenesis and mitochondrial function (Oliveira et al., 2015; Pirozzi et al., 2020). Thus, we hypothesized that SCFAs treatment might strengthen endurance capacity by increasing mitochondrial biogenesis. To test this hypothesis, mice were treated with vehicle, acetate, propionate, or butyrate for 4 weeks as indicated. Figure 6A showed SCFAs treatment did not affect body weight. Moreover, acetate and butyrate treatment significantly improved the average running time compared with the control group (Figure 6B). Furthermore, the levels of CK (Figure 6C) and BUN (Figure 6D) decreased in acetate and butyrate-treated groups, but there was no significant difference in lactic acid concentration between the four groups (Figure 6E). In addition, acetate and butyrate treatment significantly increased the mRNA expression levels of mitochondrial biogenesis-related genes in gastrocnemius compared with vehicle treatment (Figure 6F). The quantitative analysis of mitochondria DNA by RT-PCR (Figure 6G) and TEM (Figures 6H,I) revealed that the number of intermyofibrillar mitochondria in acetate and butyrate-treated groups significantly increased compared to the vehicle and propionate-treated groups. Taken together, these results suggested that acetate and butyrate could enhance endurance capacity by promoting mitochondrial biogenesis.

## Discussion

Although hypoxia exposure has been widely used to improve athletic endurance performance, the underlying mechanisms have not been fully clarified. Our results suggested that hypoxia markedly improved endurance capacity by promoting hematological parameters and muscular mitochondrial biogenesis in mice. We further demonstrated that normobaric hypoxia exposure remodeled the gut microbiota, leading to an increase in SCFAs production, which were highly correlated with endurance capacity. In addition, removing gut microbiota by antibiotic treatment abolished the effects of hypoxia on endurance capacity and muscular mitochondrial biogenesis. Moreover, the hypoxia-induced increase in the production of fecal SCFAs and enhancement of endurance capacity could be transferred to the microbiota-depleted mice by FMT. These results indicated that the gut microbiota played an essential role in the hypoxia-induced endurance capacity enhancement. Furthermore, we demonstrated that acetate and butyrate supplementation significantly increased muscular mitochondrial biogenesis, thus enhancing endurance capacity in mice. Taken together, these results demonstrated that the beneficial effects of hypoxia exposure on endurance performance were partially achieved *via* remodeling of the gut microbiota, and consequently increasing the production of intestinal SCFAs.

Previous studies have reported that hypoxia could significantly affect the gut microbiota (Bai et al., 2018; Mazel, 2019; Han et al., 2021), but the understanding of hypoxia-induced changes in the gut microbiota is far from clear. It was widely accepted that hypoxia exposure significantly decreased the aerobic bacteria and increased the anaerobic bacteria (Mazel, 2019; Han et al., 2021). However, most studies have not excluded the interference of confounding factors such as genes, diet, exercise, and hypoxia exposure model (Li and Zhao, 2015; Karl et al., 2018; Suzuki et al., 2019; Jia et al., 2020; Montoya-Ciriaco et al., 2020). Kleessen et al. (2005) reported that mountaineers exposed to high altitudes above 5,000 m have decreased beneficial *Bifidobacteria* and increased potentially pathogenic gram-negative bacteria such as *Enterobacteriaceae* that bring health risks. Moreover, strict anaerobes such as *Lactobacillus* and *Bacteroidetes* and obligate anaerobes such as *Clostridium perfringens* and *Escherichia coli* were increased in soldiers exposed to high altitudes at 3,505 m for 15 days (Adak et al., 2013). These results indicated that different levels of atmospheric oxygen might have different effects on the gut microbiota. However, little research focused on the effects of moderate altitude (2,000–3,000 m) on the gut microbiota. In addition, different from short-term exposure to severe hypoxia environment, plateau-living humans or animals showed a rich intestinal microbiota which helps the hosts to adapt to the plateau environment (Li et al., 2016, 2018, 2019; Jia et al., 2020; Zeng et al., 2021). All these studies showed the complex and varied effects of hypoxia on the gut microbiota. We conducted a randomized controlled animal study, which excluded confounding factors (including temperature, nutrition, atmospheric pressure, etc.). Our results indicated that exposure to the normobaric normoxia environment (16.4% O_2_, equivalent to an altitude of 2,000 m) for 4 weeks significantly increased the abundance of *Lactobacillus*, *Prevotellaceae_UCG-001*, *Akkermansia*, and *Bacteroides* at the genus level, accompanied by an increase of fecal acetate, propionate, and butyrate production in mice.

Moderate altitude training has emerged as a popular method to improve athletic endurance performance (Girard et al., 2013a; Millet and Brocherie, 2020). However, the underlying mechanisms have not been fully clarified (Girard et al., 2013a; Zhao et al., 2018; Millet and Brocherie, 2020; Siebenmann and Dempsey, 2020). Recent evidence indicated that exercise and the gut microbiota were interconnected (Przewłócka et al., 2020; Clauss et al., 2021). Several studies reported that endurance athletes had a higher abundance of *Prevotella* and *Akkermansia*, and a lower abundance of *Bacteroidetes* and *Lactobacillus*, accompanied by an accumulation of fecal SCFAs production (Clarke et al., 2014; Barton et al., 2018; Kulecka et al., 2020). Moreover, a single or multiple exercises could remodel the structure of the gut microbiota (Munukka et al., 2018; Scheiman et al., 2019; Kern et al., 2020). On the other hand, it was found that the metabolites of gut microbiota could participate in energy metabolism or act as an energy substrate to improve exercise performance. Frampton et al. (2020) indicated that *Veillonella* could metabolize lactate into acetate and propionate, thus improving endurance ability. In addition, Okamoto et al. (2019) suggested that acetate could act as a metabolic fuel for skeletal muscle. This study suggested that hypoxia improved endurance performance in mice by increasing the abundance of SCFA-producing bacteria, including *Akkermansia* and *Bacteroidetes*, thereby resulting in an increase of fecal SCFAs production. Moreover, the antibiotic treatment and FMT experiments suggested that the beneficial effects of hypoxia on endurance performance and SCAFs production were partly dependent on the gut microbiota. In summary, these results implied that gut microbiota might play a critical role in hypoxia enhancing endurance performance.

SCFAs, the metabolites derived from microbial fermentation of dietary fibers, have been demonstrated to regulate host energy balance, nutrition metabolism, and immune function (Koh et al., 2016; Makki et al., 2018). Accumulating evidence indicated that SCFAs participate in protein, carbohydrate, and lipid metabolism in skeletal muscle (Frampton et al., 2020). Moreover, SCFAs could enhance the athlete’s immunity, improve exercise recovery *via* anti-inflammatory activity and provide additional energy substrates for exercise performance (Frampton et al., 2020; Bongiovanni et al., 2021). Furthermore, it has been demonstrated that the muscle oxidative capacity, which is strongly correlated with the muscle mitochondrial content, was a major determinant of endurance capacity (Irrcher et al., 2003; Booth et al., 2015). Additionally, the inhibition of histone deacetylases and the activation of AMPK, PPAR-δ, and PGC-1α are likely key mechanisms through which SCFAs promote exercise performance (Frampton et al., 2020; Bongiovanni et al., 2021). In this study, we found that the contents of SCFAs were significantly positively correlated with endurance performance. Furthermore, our results indicated that that acetate and butyrate supplementation, but not propionate, promoted skeletal muscle mitochondrial biogenesis and enhanced endurance performance in mice. Taken together, our results suggested that the hypoxia-induced changes in SCFAs production contribute to the enhanced endurance capacity by promoting muscle mitochondrial biogenesis.

## Conclusion

In summary, our results have enriched the understanding of hypoxia exposure reshaping the gut microbiota. Moreover, our study suggested that the beneficial effects of the hypoxia on endurance performance were partly dependent on the increased production of SCFAs resulting from the remodeling of the gut microbiota. Overall, our study indicated that hypoxia exposure improves exercise performance partially *via* regulation of the gut microbiota-SCFAs axis.

## Data Availability Statement

The raw data of 16S rRNA sequencing was deposited in NCBI Sequence Read Archive (SRA) accession no. PRJNA778424. Available online at: https://www.ncbi.nlm.nih.gov/bioproject/PRJNA778424.

## Ethics Statement

The animal study was reviewed and approved by the Animal Care and Use Committee of the Third Military Medical University.

## Author Contributions

MM and XZ conceived and designed the study. LH, MZ, TL, and MD performed the experiments and collected the samples. LH, LZ, LY, and MZ analyzed the data. LH, JZ, XZ, and MM wrote the manuscript. MM obtained funding. All authors contributed to the article and approved the submitted version.

## Conflict of Interest

The authors declare that the research was conducted in the absence of any commercial or financial relationships that could be construed as a potential conflict of interest.

## Publisher’s Note

All claims expressed in this article are solely those of the authors and do not necessarily represent those of their affiliated organizations, or those of the publisher, the editors and the reviewers. Any product that may be evaluated in this article, or claim that may be made by its manufacturer, is not guaranteed or endorsed by the publisher.
