# Supplementary material for: Hypoxia Improves Endurance Performance by Enhancing Short Chain Fatty Acids Production via Gut Microbiota Remodeling
Source: Front Microbiol. 2022 Feb 7;12:820691. doi: 10.3389/fmicb.2021.820691 (PMC8859164; doi:10.3389/fmicb.2021.820691)
Supplement: Supplementary file 1 [file Data_Sheet_1.PDF]

Supplementary Material

1 Supplementary Figures and Tables

1.1 Supplementary Figures

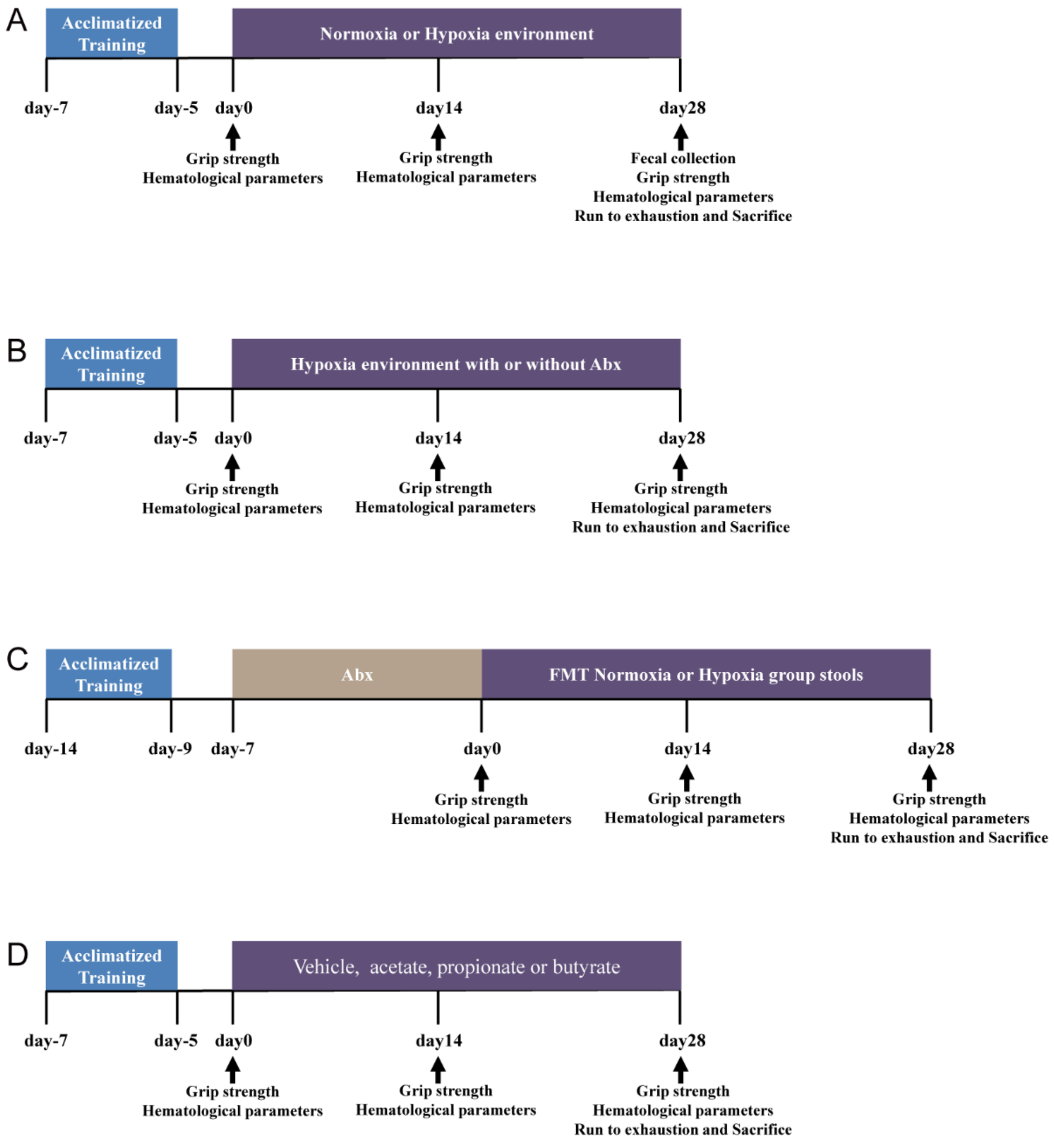

Supplementary Figure 1. Animal experimental design. **(A)** After 5 days acclimatized training, mice were exposed to normobaric normoxia (21.0 % O_2) or normobaric hypoxia (16.4 % O_2) environment for four weeks. **(B)** After 5 days acclimatized training, mice were exposed to normobaric hypoxia (16.4 % O_2) environment with or without antibiotic cocktail (Abx) for four weeks. **(C)** After 5 days acclimatized training, mice receipted Abx for one week, followed by FMT for four weeks. **(D)** After

5 days acclimatized training, mice were received 150 mM acetate (Sigma), propionate (Sigma), butyrate (Sigma) or NaCl (vehicle) in the drinking water for four weeks. Grip strength and hematological parameters were measured at 0w, 2w, and 4w.

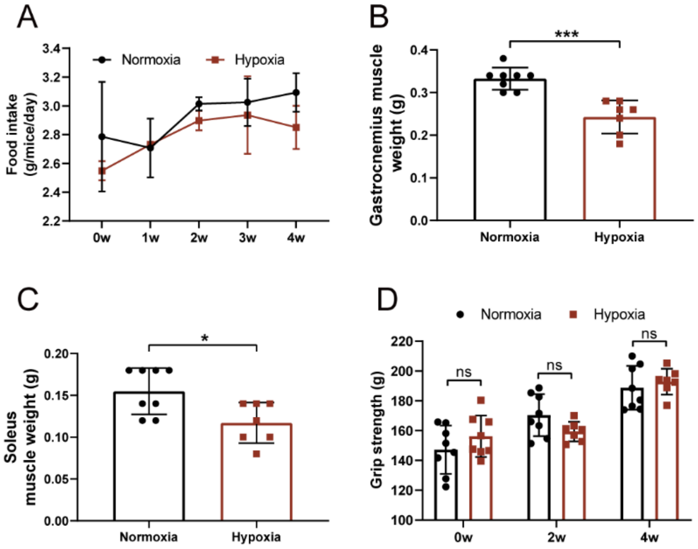

Supplementary Figure 2. (A) Food intake was measured once a week. The gastrocnemius **(B)** and soleus muscle **(C)** weights were measured. **(D)** Grip strength was measured as indicated at 0w, 2w, and 4w. Data are presented as means ± SD. * $p < 0.05$, ** $p < 0.01$, *** $p < 0.001$ (Student's t-test).

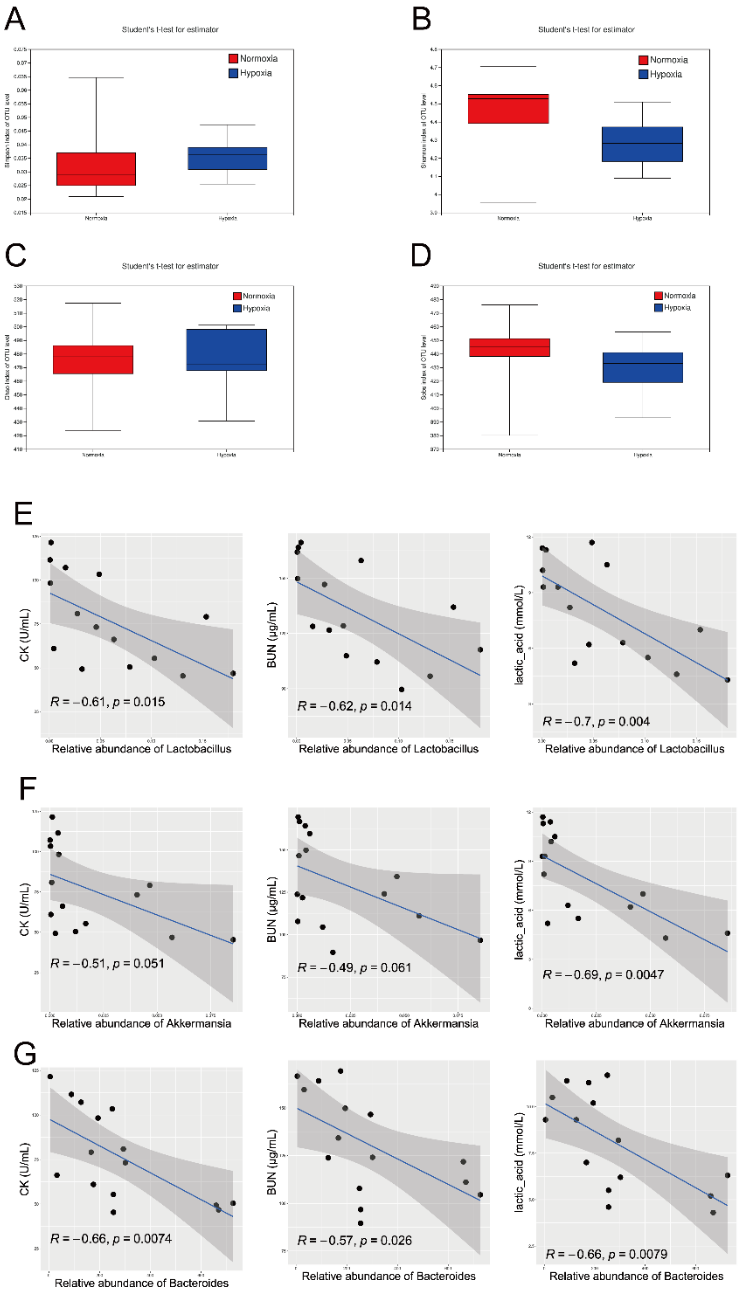

Supplementary Figure 3. The α-diversity was assessed by the Simpson index **(A)**, Shannon index **(B)**, Chao index **(C)**, and Sobs index **(D)** (at the OTU level). **(E)** Plots show the relationship between *Lactobacillus* abundance and CK, BUN, and lactic acid represented as a smoothing spline with a 95% confidence interval (shaded region). **(F)** Plots show the relationship between *Bacteroides* abundance and CK, BUN, and lactic acid represented as a smoothing spline with a 95% confidence interval (shaded region). **(G)** Plots show the relationship between *Akkermansia* abundance and CK,

BUN, and lactic acid represented as a smoothing spline with a 95% confidence interval (shaded region). (A-D, Student's t-test; E-G, Pearson's correlation).

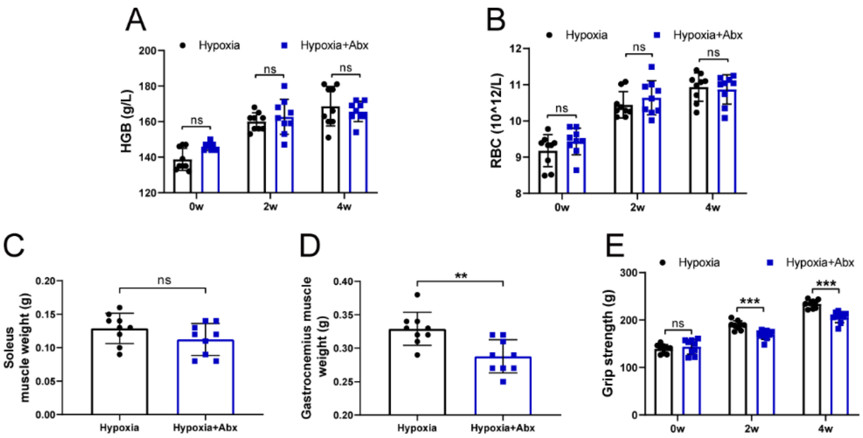

Supplementary Figure 4. HGB **(A)** and RBC **(B)** in the tail blood were detected as indicated. Soleus **(C)** and gastrocnemius **(D)** muscles weight and grip strength **(E)** were measured. Data are presented as means ± SD. * $p < 0.05$, ** $p < 0.01$, *** $p < 0.001$ (Student's t-test).

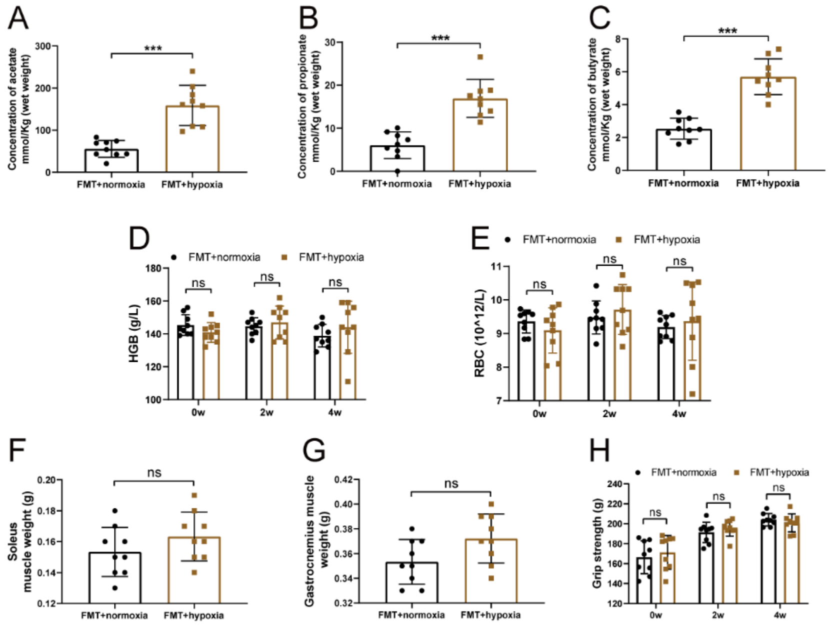

Supplementary Figure 5. The contents of acetate **(A)**, propionate **(B)**, and butyrate **(C)** were measured by GC–MS in cecal contents. HGB **(D)** and RBC **(E)** in the tail blood were detected as

indicated. Soleus **(F)** and gastrocnemius **(G)** muscles weight and grip strength **(H)** were measured. Data are presented as means ± SD. * $p < 0.05$, ** $p < 0.01$, *** $p < 0.001$ (Student's t-test).

1.2 Supplementary Tables

Table S1. Primers used in this study.

The figure legends are required to have the same font as the main text, 12 point normal Times New Roman, single spaced. Please use a single paragraph for each legend and prepare the figures keeping in mind the PDF layout.

	Forward primers	Reverse primers
PGC-1α	GTCCTTCCTCCATGCCTGAC	GACTGCGGTTGTGTATGGGA
Tfam	TGGGGACCAGGGCAGCCATT	AGGGCTTGCTGCCCACACAT
VEGF	ACCCTGGCTTTACTGCTGTACCT	TCATGGGACTTCTGCTCTCCTT
COX2	TTTTCAGGCTTCACCCTAGATGA	GAAGAATGTTATGTTATGTTTACTCCTA
18s rRNA	TAGAGGGACAAGTGGCGTTC	CGCTGAGCCAGTCAGTGT
β-actin	GTGACGTTGACATCCGTAAAGA	GCCGGACTCATCGTACTCC
