# Supplementary figures and images for: Hypoxia Improves Endurance Performance by Enhancing Short Chain Fatty Acids Production via Gut Microbiota Remodeling
Source: Front Microbiol. 2022 Feb 7;12:820691. doi: 10.3389/fmicb.2021.820691 (PMC8859164; doi:10.3389/fmicb.2021.820691)

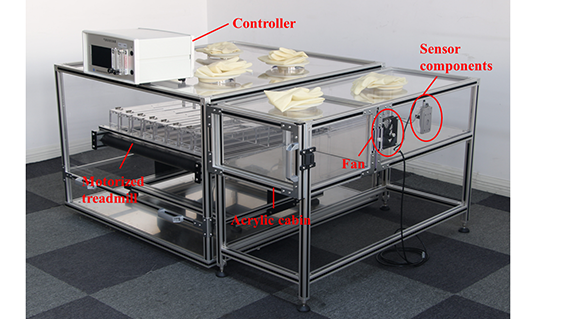

Supplement: Supplementary file 2 [file Image_6.TIF]
